# The Contributions of Memory and Vocabulary to Non-Verbal Ability Scores in Adolescents with Intellectual Disability

**DOI:** 10.3389/fpsyt.2016.00204

**Published:** 2016-12-27

**Authors:** Chantanee Mungkhetklang, Edith L. Bavin, Sheila G. Crewther, Nahal Goharpey, Carl Parsons

**Affiliations:** ^1^School of Psychology and Public Health, La Trobe University, Melbourne, VIC, Australia; ^2^Port Phillip Specialist School, Port Melbourne, VIC, Australia

**Keywords:** intellectual disability, non-verbal IQ tests, short-term memory, working memory, vocabulary

## Abstract

It is usually assumed that performance on non-verbal intelligence tests reflects visual cognitive processing and that aspects of working memory (WM) will be involved. However, the unique contribution of memory to non-verbal scores is not clear, nor is the unique contribution of vocabulary. Thus, we aimed to investigate these contributions. Non-verbal test scores for 17 individuals with intellectual disability (ID) and 39 children with typical development (TD) of similar mental age were compared to determine the unique contribution of visual and verbal short-term memory (STM) and WM and the additional variance contributed by vocabulary scores. No significant group differences were found in the non-verbal test scores or receptive vocabulary scores, but there was a significant difference in expressive vocabulary. Regression analyses indicate that for the TD group STM and WM (both visual and verbal) contributed similar variance to the non-verbal scores. For the ID group, visual STM and verbal WM contributed most of the variance to the non-verbal test scores. The addition of vocabulary scores to the model contributed greater variance for both groups. More unique variance was contributed by vocabulary than memory for the TD group, whereas for the ID group memory contributed more than vocabulary. Visual and auditory memory and vocabulary contributed significantly to solving visual non-verbal problems for both the TD group and the ID group. However, for each group, there were different weightings of these variables. Our findings indicate that for individuals with TD, vocabulary is the major factor in solving non-verbal problems, not memory, whereas for adolescents with ID, visual STM, and verbal WM are more influential than vocabulary, suggesting different pathways to achieve solutions to non-verbal problems.

## Introduction

Cognitive ability in children is most often assessed with the Wechsler Intelligence Scale for Children (WISC) (1). However, there are many situations where the language-based WISC could be argued to be less appropriate, e.g., when testing hearing and language impaired individuals and those with neurodevelopmental disorders, including intellectual disability (ID) (2) and/or autism spectrum disorder (ASD) (3), attention deficit/hyperactive disorder (4), or specific learning disorder (5). This has led to suggestions that non-verbal intelligence tests such as the Raven’s colored progressive matrices (RCPM) (6), the test of Non-verbal Intelligence-Fourth Edition (TONI-4) (7), the Comprehensive Test of Non-verbal Intelligence-Second Edition (8), the Leiter International Performance Scale-Revised (Leiter-R) (9), and the Wechsler Non-verbal Scale of Ability (WNV) (10) may be more appropriate. However, to date, there has been little research investigating the role verbal ability plays in visual problem solving tasks included in these tests or whether these tests adequately assess the cognitive abilities of individuals with ID, whose language is often impaired.

Intellectual disability is associated with delayed early development and severe problems with adaptive functioning (11), typically associated with limited cognitive abilities (12–15). Currently, the most common diagnostic criterion for ID is an estimated intelligence Quotient (IQ) below 70 on the Wechsler Intelligence Scale for Children-Fourth Edition (WISC-IV) (11, 16, 17). Interestingly, despite the fact that the performance subtests from the WISC assume a degree of language for the child to understand and remember what the task requires and to respond appropriately (1, 18, 19), there has been comparatively little research investigating the impact of language and vocabulary on scores from IQ measures.

Wechsler reportedly viewed verbal and performance tests as equally valid measures of intelligence despite the fact that the Full Scale IQ score for the WISC is a combination of expressive vocabulary tasks and non-verbal problem solving (20). Non-verbal tests, used as alternative assessment tools of IQ, are assumed not to measure verbal skills, although the content and administration method of the non-verbal intelligence tests varies considerably (21–25), and verbal instructions are often used. Some researchers have found that language influences the scores of non-verbal IQ tests (15, 26) and a significant association between performance on the Wisconsin Card Sorting Test and language measures (comprehension and naming) have been reported (27). In other research (28), both correct performance and error types on the RCPM were associated with receptive vocabulary scores from the Peabody Picture Vocabulary Test (PPVT).

While there has been previous research with children with ID that has included receptive and expressive language measures [e.g., Ref. (15, 29, 30)], relatively little attention has been paid to directly exploring the unique contribution of vocabulary scores (expressive and receptive) to the non-verbal ability of individuals with ID.

Vocabulary development has been shown to be associated with verbal (phonological) short-term memory (STM) (31–33) and, as argued by Gathercole and Alloway (34) verbal STM is particularly important in early vocabulary development, when there is little available support from existing lexical knowledge, which indicates the separability of vocabulary and STM (31). This view was supported in a study with children with down syndrome (35). However, while there has been research on STM in ID [e.g., Ref. (36, 37)], the unique variance contributed by expressive and receptive vocabulary to non-verbal IQ scores in addition to that contributed by STM (visual and spatial) and working memory (WM) has not been previously been examined. Identifying the unique contributions of memory and vocabulary to non-verbal assessment results will add to our understanding of the performance of individuals with ID on Non-verbal IQ tests. In previous research, IQ, STM, and WM have been shown to be associated (34, 38–41), but they contribute *differentially* to cognitive abilities (41–44), as illustrated by the reported correlation coefficients between WM and performance on tests of overall cognitive ability; they range from approximately 0.60 to 0.90.

There has been discussion in the literature of the phonological and visuospatial memory problems that individuals with ID seem to have with WM tasks (45), and deficits in both visual and verbal WM have been shown to increase with the severity of ID (46). Of interest is that visual facilitators (pictures and visual schematics) have been found to usefully assist development of more efficient memory protocols for individuals with ASD and ID (34), suggesting that visual processing ability compensates for deficits in verbal processing.

In the current study, we tested a group of adolescents with ID and a group of children with typical development (TD) to investigate the extent to which expressive and receptive vocabulary scores add to the variance contributed by STM and WM to their non-verbal IQ scores. Three tests, the RCPM, the TONI-4, and the WNV, were chosen on the basis of the visual problem solving content and limited requirements for verbal expression (either in administration and/or responses). All three tests were designed to have a low degree of cultural loading and linguistic demands (22), and scores on these three tests have been found to provide a similar mental age as the WISC-IV for 5–7-year-old children with TD and adolescents with ID aged 11–16 years (47).

The specific aims of the study were to enhance understanding of the non-verbal ability of adolescents with ID. We compared a group of individuals with ID and a group of children with TD of comparable mental age in order to determine the following:

- The amount of variance contributed to non-verbal test scores by auditory and visual STM and WM.
- The amount of additional variance contributed to non-verbal scores by expressive and receptive vocabulary scores.

## Materials and Methods

### Participants

The 56 participants were 17 adolescents with ID ranging in age from 11 years 4 months to 16 years 7 months (5 females, 12 males, M = 13.57, SD = 1.28) and 39 children with TD ranging in age from 5 years 4 months to 7 years 11 months (16 females, 23 males, M = 6.35, SD = 0.76). Of the ID participants, eight had a diagnosis of ASD, three had attention deficit and hyperactive disorder, two had idiopathic ID, and one had Williams syndrome; and three had genetic disorder diagnoses, i.e., congenital occipital encephalocele, galactosemia (thalamic stroke), and fetal anticonvulsant syndrome. None of the children with TD had a diagnosis of any neurological or cognitive disorder. We compared the ID group with a group of school children with TD aged 5–7 years, an age expected to be equivalent to the mean mental age of the ID group.

Parents/guardians provided informed consent prior to their child’s participation. All individuals were screened for normal hearing and vision. Ethics approval was obtained from the La Trobe University Human Ethics Committee (HEC approval number: HEC06-036 and FHEC13/R22) and the State Department of Education (Approval number: 2012-001425). Permission to conduct testing in the schools was obtained from the school principals. Parents/guardians provided informed consent prior to their child’s participation.

### Materials

#### Non-Verbal Tests

##### Raven’s Colored Progressive Matrices

The RCPM is a standardized test of non-verbal intelligence. The RCPM measures fluid ability, that is, the ability to form comparisons, to reason by analogy, and to organize spatial perceptions into a systematically related whole. It was developed for use with individuals with TD aged 5 years 11 months to the elderly, as well as the mentally and physically impaired. Each item consists of an array of colored patterns with a missing portion, and the examinee is required to choose the missing elements from a group of six possible options. Split-half reliabilities range from 0.65 to 0.94. Concurrent validity coefficients between Raven’s progressive matrices and other intelligence tests are in the 0.50 to 0.80 s (6).

##### Test of Non-Verbal Intelligence-Fourth Edition

The TONI-4 is a standardized test of non-verbal intelligence. Non-verbal items of the standard figures matrices type were used to develop the TONI as an assessment of the cognitive ability of children and adults. The assessment is designed for use with individuals aged 6 years 0 months to 89 years 11 months. Each item is a black and white pattern with a missing portion. Items are in a multiple choice format, with either five or six response alternatives. Instructions are given *via* pantomime; individuals respond by pointing, nodding, or blinking. Internal consistency reliability ranges from 0.94 to 0.97. Correlation coefficients with other non-verbal intelligence tests range from 0.73 to 0.79 (48).

##### Wechsler Non-Verbal Scale of Ability

The WNV is a standardized test of non-verbal intelligence. It is designed for use with TD individuals aged 4 years 0 months to 21 years 11 months. The WNV comprises seven subtests, the particular ones administered depending on the age of the examinee: 4 years 0 month to 7 years 11 months or 8 years 0 months to 21 years 11 months. The WNV subtests for the younger age group were used in this study, that is, Matrices, Coding A, Object Assembly, and Recognition. The Matrices subtest requires the examinee to identify how different geometric shapes are spatially or logically interrelated and to complete the relationship among the parts. The test items are varied (e.g., geometric pattern, reasoning by analogy, and spatial visualization). The displays use basic geometric figures, such as squares, circles, and triangles, and use some combination of colors: black, white, yellow, blue, and green. The Coding subtest requires the examinee to co-operate with verbal instructions to copy symbols (e.g., dash, two vertical lines, and an open parenthesis) that are paired with simple geometric shapes according to a key provided at the top of the page. The Object assembly subtest consists of items that require the examinee to complete pieces of a puzzle to form a recognizable object such as a ball or a car. The Recognition subtest requires the examinee to examine a stimulus (e.g., a square with a small circle in the center) for 3 s and then choose which of four or five options is identical to the stimulus that was just seen. Average internal consistency reliability for the Full Scale IQ on four subtests versions are 0.91. Correlations between the four-subtest version Full Scale IQ and other test of intelligence range between 0.71 and 0.82 (10).

#### Vocabulary Tests

##### Peabody Picture Vocabulary Test-Fourth Edition (PPVT-4)

We used the PPVT-4 to measure receptive vocabulary. It is used for individuals aged 2 years 6 months to 90+ years. The examinee is required to choose from a set of four pictures, the one that best relates to the word said aloud by the examiner. Correlation coefficients with the Expressive Vocabulary Test-Second Edition (EVT-2) average 0.82 and with other tests range from 0.41 to 0.79 (49).

##### Expressive Vocabulary Test-Second Edition

The EVT-2 was used as the assessment of expressive vocabulary. It is designed for individuals aged 2 years 6 months to 90+ years old. The examinee labels the pictures aloud, e.g., when shown a picture of a ball, the expected answer is “ball.” Correlation coefficients with the PPVT-4 scale average 0.82 and with other tests range from 0.50 to 0.81 (50). The PPVT and EVT-2 are co-normed, allowing direct comparisons between their respective standard scores.

#### Memory Tasks

##### Digit Span Visual and Verbal Tasks

Our measure of STM was forward digit span. Backward digit span, which requires manipulation of the information presented, was the measure of WM. In order to establish consistent presentation of the stimuli, a VPixx computer program (51) was developed to present the Digit Span tasks—with both visual and verbal presentations of numbers. Participants were required to complete both visual and verbal version of the tasks. The order in which the tasks were given was counterbalanced. For the verbal tasks, participants listened to the numbers presented through the computer sound system at a rate of 1/s. Two trials per span length (i.e., the number of digits in the given sequence) were provided; when two trials of the same span length were answered incorrectly testing was discontinued. For the visual task, a list of digits (1–9) was presented on a computer screen at a rate of 1/s.

### Procedure

The participants were tested individually at school. All tests were conducts using standardized instructions, scoring, and interpretation. The results for each participant were considered a valid assessment, not impeded by behavioral or emotional factors. Test sessions lasted 15–45 min, depending on each participant’s concentration span.

### Analysis

To compare groups on their scores for each of the non-verbal assessments, the two vocabulary and four memory measures, one-way ANOVAs between groups were used. A regression analysis for each non-verbal test identified the amount of variance contributed by each of the memory measures. In a second series of regression analyses, the vocabulary scores were added (together with the memory scores) to identify the additional variance contributed by expressive and receptive vocabulary to each of the three non-verbal IQ test scores.

## Results

Scores from each non-verbal test and the PPVT and EVT were first checked by group for normality; the results met all expected criteria. Descriptive statistics (the mean and SD of the raw scores for each test) for the three non-verbal tests for each group are reported in Table 1.

**Table 1:** **Descriptive statistics and raw scores for the TD and ID groups**.

		TD (*n* = 39)		ID (*n* = 17)	
		Mean (range)	SD	Mean (range)	SD
Non-verbal tests	RCPM	24.49 (11–35)	5.46	25.71 (13–32)	6.94
	TONI-4	24.08 (14–36)	5.46	22.00 (9–34)	6.25
	WNV	99.53 (67.5–146.5)	19.22	98.26 (36.5–144.0)	36.70
Vocabulary tests	EVT-2	101.03 (72–123)	11.14	88.47 (58–119)	15.92
	PPVT-IV	127.15 (95–153)	16.08	123.29 (79–175)	26.56
Digit span forward (STM tasks)	Visual	3.97 (2–6)	0.96	3.65 (0–5)	1.37
	Verbal	4.28 (3–6)	0.76	4.18 (2–6)	1.18
Digit span backward (WM tasks)	Visual	3.18 (1–5)	0.85	2.65 (0–4)	0.93
	Verbal	3.21 (2–5)	0.86	2.76 (0–5)	1.35

*RCPM, Raven’s colored progressive matrices; TONI-4, Test of Non-Verbal Intelligence-Fourth Edition; WNV, Wechsler Non-Verbal Scale of Ability; EVT-2, Expressive Vocabulary Test-Second Edition; PPVT-IV, Peabody Vocabulary Test-Fourth Edition; STM, short-term memory; WM, working memory; TD, typically developing group; ID, intellectual disability group*.

One-way ANOVAs between groups were conducted on scores from each of the three non-verbal tests. There were no significant group differences on scores for the RCPM, *F* (1, 54) = 0.498, *p* = 0.483, Cohen’s *d* = 0.19, nor for the TONI-4, *F* (1, 54) = 1.564, *p* = 0.216, Cohen’s *d* = 0.35, nor for the WNV, *F* (1, 54) = 0.029, *p* = 0.866, Cohen’s *d* = 0.04. The ID and TD groups performed similarly on each of the three tests, indicating that the non-verbal mental age of each group was similar, as expected. However, there was a large effect size for the TONI-4.

One-way ANOVAs were also conducted on the expressive and receptive vocabulary test scores to compare performance of the ID and TD groups. Although there were no significant group differences on the PPVT-IV raw scores, *F* (1, 54) = 0.451, *p* = 0.505, Cohen’s *d* = 0.17, there was a significant group difference on the EVT-2 raw scores, *F* (1, 54) = 11.48, *p* = 0.001, Cohen’s *d* = 0.91. The EVT-2 scores were lower for the ID group than the TD group.

For the visual and verbal digit span forward and backward tasks, the TD group mean scores were higher than for the ID group. One-way ANOVAs between groups were conducted on each task. There was a significant group difference on the digit span backward visual task, *F* (1, 54) = 4.354, *p* = 0.042, Cohen’s *d* = 0.59, with a large effect size. However, there were no significant group differences for digit span forward visual, *F* (1, 54) = 1.056, *p* = 0.309, Cohen’s *d* = 0.27, digit span forward verbal, *F* (1, 54) = 0.161, *p* = 0.690, Cohen’s *d* = 0.10, digit span backward verbal, *F* (1, 54) = 2.160, *p* = 0.147, Cohen’s *d* = 0.40, although the effect size for the latter was large.

Regression analyses on the scores from the three non-verbal tests were first conducted with the two STM scores as predictor variables, that is, visual STM and verbal STM. In a second regression analysis, the two WM tasks were included as predictor variables. Table 2 shows the results. For the TD group, visual and verbal STM did not contribute significant variance to any of the three non-verbal test scores. Visual STM contributed only ~7% to the RCPM and the WNV and ~3% to the TONI-4; verbal STM contributed ~3% to the RCPM, ~7% to the TONI-4, and ~9% to the WNV. In contrast, visual WM contributed significant variance to two of the non-verbal tests: 10.5 and 12.5%, respectively, to the TONI-4 and the WNV, but no significant variance to the RCPM (~5%). By comparison, verbal WM contributed significant variance to the RCPM (10.5%), but not to the TONI-4 (~3%) and WNV (~7%).

**Table 2:** **Results from the multiple regression analysis using memory scores for TD group and ID group**.

		TD (*n* = 39)						ID (*n* = 17)					
		RCPM		TONI-4		WNV		RCPM		TONI-4		WNV	
	
		*R*^2^ change	Partial^2^	*R*^2^ change	Partial^2^	*R*^2^ change	Partial^2^	*R*^2^ change	Partial^2^	*R*^2^ change	Partial^2^	*R*^2^ change	Partial^2^
STM	Visual	7.8	0.528	3.3	0.425	7.8	0.529	46.2**	0.825	29.3*	0.735	32.6*	0.755
	Verbal	3.5	0.433	7.1	0.517	9.8	0.558	14.4	0.616	9.7	0.558	2.4	0.392
WM	Visual	5.2	0.478	10.5*	0.569	12.5*	0.594	23.6*	0.696	21.3	0.679	21.7	0.683
	Verbal	10.5*	0.569	3.3	0.424	7.2	0.518	34.4*	0.760	26.9*	0.720	25.0*	0.707

*RCPM, Raven’s colored progressive matrices; TONI-4, Test of Non-verbal Intelligence-Fourth Edition; WNV, Wechsler Non-verbal Scale of Ability; STM, short-term memory; WM, working memory; TD, typically developing group; ID, intellectual disability group*.

*p < 0.01.

**p < 0.001.

For the ID group, visual STM contributed significant variance to all three non-verbal test scores: 46.2% to the RCPM, 29.3% to the TONI-4, and 32.6% to the WNV, but as for the TD group, verbal STM did not contribute significant variance to any of the non-verbal test scores. In contrast to the TD results, visual WM contributed significant variance (23.6%) to the RCPM, but not to the TONI-4 (21.3%) or the WNV (21.7%). In addition, verbal WM contributed significant variance to all three non-verbal test scores: 34.4% to the RCPM, 26.9% to the TONI-4, and 25% to the WNV.

In order to determine the amount of variance contributed by Vocabulary over and above that contributed by Memory, the Vocabulary scores were added as predictor variables in a second set of regression analyses. As shown in Table 3, for the TD group with EVT-2 scores added, for all four memory measures there was a significant increase in the variance. Approximately two to three times more variance was found than previously to the RCPM and the TONI-4, and even more to the WNV. The unique variance for expressive vocabulary was greater than for memory for all three non-verbal tests. By comparison, for the ID group while the EVT-2 scores increased the variance for all three non-verbal test scores, with visual STM, vocabulary contributed significant unique variance for the RCPM and WN. In contrast to the TD group, when EVT-2 scores were included with verbal STM, no significant unique variance was contributed by vocabulary. With visual WM and EVT-2 scores, expressive vocabulary contributed significant unique variance only to the RCPM; a similar result was found when EVT-2 scores and verbal WM were included in the regression. The unique variance for memory for the ID group was larger than for vocabulary in all four analyses.

**Table 3:** **Variance contributed to non-verbal test scores with vocabulary in addition to memory scores**.

			TD (*n* = 39)						ID (*n* = 17)					
Vocab.	Memory		RCPM		TONI-4		WNV		RCPM		TONI-4		WNV	
	
			*R*^2^ change	Partial^2^	*R*^2^ change	Partial^2^	*R*^2^ change	Partial	*R*^2^ change	Partial^2^	*R*^2^ Change	Partial^2^	*R*^2^ change	Partial^2^
EVT-2	STM	Visual	21.3*	0.395^a^	20.3*	0.192^a^	36.4**	0.327^a^	57.9**	0.826^a^	29.4	0.728^a^	40.0*	0.743^a^
				0.619^b^		0.648^b^		0.746^b^		0.683^b^		0.217^b^		0.576^b^
		Verbal	19.5*	0.210^a^	21.7*	0.366^a^	36.9**	0.377^a^	31.7	0.606^a^	10.7	0.548^a^	15.0	0.332^a^
				0.637^b^		0.629^b^		0.741^b^		0.671^b^		0.322^b^		0.596^b^
	WM	Visual	19.9*	0.286^a^	23.2**	0.438^a^	37.8**	0.425^a^	38.1*	0.681^a^	21.6	0.670^a^	30.5	0.663^a^
				0.627^b^		0.614^b^		0.733^b^		0.660^b^		0.255^b^		0.578^b^
		Verbal	26.7**	0.549^a^	21.8*	0.374^a^	39.5**	0.493^a^	42.2*	0.719^a^	27.1	0.712^a^	29.7	0.656^a^
				0.652^b^		0.662^b^		0.768^b^		0.587^b^		-0.217^b^		0.500^b^
PPVT-IV	STM	Visual	14.8	0.425^a^	19.0*	0.192^a^	39.8**	0.295^a^	62.9**	0.840^a^	39.9*	0.727^a^	44.1*	0.752^a^
				0.524^b^		0.635^b^		0.767^b^		0.747^b^		0.614^b^		0.643^b^
		Verbal	12.5	0.288^a^	20.6*	0.379^a^	40.6**	0.375^a^	37.2**	0.624^a^	25.3	0.550^a^	19.3	0.360^a^
				0.551^b^		0.617^b^		0.764^b^		0.718^b^		0.624^b^		0.645^b^
	WM	Visual	13.6	0.375^a^	23.0**	0.475^a^	42.4**	0.473^a^	30.8	0.507^a^	23.4	0.549^a^	24.8	0.538^a^
				0.545^b^		0.612^b^		0.765^b^		0.555^b^		0.406^b^		0.446^b^
		Verbal	19.1*	0.535^a^	20.0*	0.345^a^	42.0**	0.457^a^	43.3*	0.696^a^	31.1	0.653^a^	30.9	0.631^a^
				0.557^b^		0.646^b^		0.783^b^		0.607^b^		0.489^b^		0.528^b^

*RCPM, Raven’s colored progressive matrices; TONI-4, Test of Non-Verbal Intelligence-Fourth Edition; WNV, Wechsler Non-Verbal Scale of Ability; EVT-2, Expressive Vocabulary Test-Second Edition; PPVT-IV, Peabody Vocabulary Test-Fourth Edition; STM, short-term memory; WM, working memory; TD, typically developing group; ID, intellectual disability group*.

*^a^Partial^2^ for Memory scores*.

*^b^Partial^2^ for Vocabulary scores*.

*p < 0.01.

**p < 0.001.

The addition of PPVT scores rather than EVT-2 scores for the TD group showed a similar pattern overall as for the EVT-2. The unique variance contributed by the PPVT was greater than for Memory. With visual STM significant unique variance from receptive vocabulary was found for the Toni and WVN but not for the RCPM. With verbal STM and also with visual WM, similar results were found. However, for verbal WM, receptive vocabulary contributed significant unique variance to all three non-verbal scores. For the ID group, with visual STM, significant unique variance was contributed by receptive vocabulary to all three non-verbal tests but more unique variance was contributed by Memory than Vocabulary. With verbal STM, a significant contribution from receptive vocabulary was found only for the RCPM. With visual WM, no significant contribution from receptive vocabulary was found for any of the three non-verbal tests, but with verbal WM there was a significant contribution from receptive vocabulary for the RCPM. That is, vocabulary scores added to the variance when they were included with the memory scores, but the variance contributed differed for each of the three non-verbal tests.

## Discussion

The results overall provide evidence that memory (both STM and WM) and vocabulary (both expressive and receptive vocabulary) are associated with performance on non-verbal tests of IQ, both for children with TD and adolescents with ID of the same non-verbal mental age.

As expected there were no significant group differences on the three non-verbal test scores, although the mean scores of the ID group were slightly higher, and there was greater variability on all three non-verbal tests for the ID group than for the TD group and with a large effect size on the TONI. Nor was there a significant group difference for receptive vocabulary, but the expressive vocabulary scores differed with the younger, TD group achieving significantly higher scores than the adolescent ID group. For the memory tasks, there was a group difference on the visual WM task; given the large effect sizes on both WM tasks it is likely that with a larger sample of individuals with ID, significant group differences would have been found for both the verbal and visual WM tasks.

No evidence for a significant relationship between STM and non-verbal problem solving was found for the TD group, whereas visual STM contributed significantly to all three non-verbal test scores for the ID group. This indicates that the participants with ID were more likely to be successful in problem solving on the non-verbal tasks if they had higher visual STM scores and that visual STM played a major role in their problem solving strategy.

In contrast, both visual WM and verbal WM contributed variance to the three non-verbal tests for the TD group but differently depending on the test involved. For solving the RCPM tasks, verbal WM was important. This indicates that the young children were rehearsing as they identified features of the patterns they needed to complete and held these in memory as they compared the alternatives in order to reach a decision on how best to complete the matrices. For the other two non-verbal tests, visual WM was more important for solving the problems. However, for older children than in our study (i.e., over 7 years), verbal WM is likely to be more involved since memory develops over the primary school years (52, 53).

Both visual WM and verbal WM played a role in solving the RCPM tasks for the ID group, but for solving tasks on the other two non-verbal tests verbal WM but not visual WM contributed significantly. Based on these results, non-verbal assessments cannot be viewed as “language free.” Success on solving the problems for individuals with ID depends on using their verbal WM. Of interest is that for both groups receptive and expressive vocabulary scores added significant variance to that contributed by memory to the non-verbal test scores for both groups but less so for the ID group.

Our findings clearly show that vocabulary (expressive and receptive) will impact on how well a child with TD in the early school years performs on non-verbal ability tests. The results show a greater contribution from vocabulary than memory when they are solving non-verbal problems. The WNV, in particular, showed a greater influence of vocabulary for the TD group. Four tasks of a different nature, drawing on different cognitive skills, make up the assessment, and we assume this had some influence on the result. In contrast, the results for the ID group support the view that visual STM as well as visual and verbal WM play a more important role than vocabulary in completing non-verbal tasks for adolescents with ID. Visual STM had a greater influence than verbal STM or WM (visual or verbal) on their non-verbal scores.

The study contributes to the literature by comparing performance on three non-verbal tests and considering the unique contributions of memory and vocabulary to the assessment results. However, the study is not without limitations. The ID participants were all recruited from one special school, and there were multiple diagnoses. The sample size was not large and could have been increased by extending recruitment to other schools. Even so, significant results were found. However, the results need to be replicated with larger samples from different educational environments. In past research testing intelligence, memory and language, principal components analysis identified that more variance loaded onto a fluid reasoning factor (i.e., higher order thinking skills) for younger children with TD than older children (31). Thus, while we were interested in comparing groups of similar mental age, inclusion of a TD group of comparable chronological age as the ID group could have provided valuable information about the roles of memory and vocabulary in problem solving for tasks included in non-verbal assessments of IQ.

## Conclusion

Our study has demonstrated that both TD and ID individuals draw on different abilities to achieve the same result when solving non-verbal problems. The TD group drew more on their vocabulary knowledge, while the ID apparently relied more on visual memory. Even though the ID were chronologically much older and likely to have had greater experience of language, when compared with the TD group their expressive vocabulary was significantly lower. Thus, adolescents with ID are likely to achieve low scores on assessments (including non-verbal assessments) that may or may not use verbal instructions or require verbal responses. Our current findings indicate that more research on the interface between memory and language skills in relation to cognitive ability will be beneficial in helping develop appropriate ways of assessing the cognitive abilities and development of individuals with ID. In additions, a focus on training visual STM for young people with ID is recommended.

## Author Contributions

CM collected the data, conducted the analyses and interpretation of the data, and prepared early drafts of the paper. SC and EB conceptualized and supervised development of the research, assisted with analysis and interpretation of the data, and contributed to writing the manuscript. NG participated in the collection of the data and helped manuscript preparation. CP helped in recruitment and provide testing facilities. All the authors read and approved the final manuscript.

## Conflict of Interest Statement

The authors declare that the research was conducted in the absence of any commercial or financial relationships that could be construed as a potential conflict of interest.
